# Printability of Nixtamalized Corn Dough during Screw-Based Three-Dimensional Food Printing

**DOI:** 10.3390/foods13020293

**Published:** 2024-01-17

**Authors:** Verónica Valeria Rodríguez-Herrera, Takumi Umeda, Hiroyuki Kozu, Tomoko Sasaki, Isao Kobayashi

**Affiliations:** 1 Institute of Food Research, National Agriculture and Food Research Organization, Tsukuba 305-8642, Japan; s2330335@u.tsukuba.ac.jp (V.V.R.-H.); umedat307@affrc.go.jp (T.U.); tomokos@affrc.go.jp (T.S.); 2 Graduate School of Science and Technology, University of Tsukuba, Tsukuba 305-8577, Japan; 3 School of Integrative and Global Majors (SIGMA), University of Tsukuba, Tsukuba 305-8577, Japan

**Keywords:** extrusion-based 3D food printing, corn-based dough, printability, extrudability, shape retention, rheological properties, texture

## Abstract

This study aimed to analyze the printability of corn-based dough during screw-based three-dimensional (3D) food printing (3DFP) by relating its rheological and mechanical properties to its screw-based 3DFP performance, with the objective of providing insights into the utilization of corn-based dough to produce 3D-printed foods. Screw-based 3DFP was performed using seven corn-based doughs with different nixtamalized corn flour (NCF) and water contents. Afterward, their rheological and mechanical properties were analyzed and associated with their screw-based 3DFP performance. The results showed that stable printability was obtained within a specific range of NCF content in the dough (30–32.5 wt%). Below this range, the 3D-printed foods flattened, while above it, the extrudability of the dough was affected. The printability of the dough was influenced by different rheological and mechanical properties, depending on the stage of the screw-based 3DFP process. During the extrusion stage, the loss tangent at nozzle strain, yield stress, apparent viscosity, and adhesiveness mainly affected the extrudability of the dough. In contrast, the loss tangent at minimum strain, elastic modulus, Young’s modulus, and hardness influenced the self-supporting stage. Therefore, it is important to find a balance between all of these properties, where stable extrudability and self-supporting of the 3D structure are achieved.

## 1. Introduction

Three-dimensional (3D) food printing (3DFP) is a novel technology for producing food and is gaining interest and popularity because it permits the design and manufacture of complex food products with particular textures, tastes, structures, and compositions [1,2]. This technology enables the customization of food products for different groups, such as aged individuals [3], athletes, or kids [4] with special nutritional needs. Furthermore, this technology allows the incorporation of low-quality fruits and vegetables [5], low-value byproducts, and low-carbon food ingredients, such as algae [6], insects [7], and alternative proteins [8], as raw materials, thereby reducing food waste and environmental impact and increasing the number of available food materials [1].

Three-dimensional food printing (3DFP) refers to the action of making food products from a 3D digital model by depositing layers of food materials one on top of the other until completion of the designed shape [9]. Currently, four techniques are being used to perform 3DFP: extrusion-based 3DFP, selective laser sintering, binder jetting, and inkjet printing [10]. Among them, extrusion-based 3DFP is the most widely used method [1].

Extrusion-based 3DFP can be performed through three different mechanisms: air pressure, screw extrusion, and syringe extrusion [11]. During the air pressure mechanism, pneumatic force is used to push food materials through a nozzle. During syringe extrusion, a piston is gradually pushed down by a motor, causing the extrusion of food materials. In screw extrusion, a screw is rotated by a motor to transport food ingredients through a nozzle.

The success of the printing process depends on the printability of food materials, printing conditions, and the size and shape of the designed 3D model [10]. Printability consists of two main independent components: extrudability and shape stability. Extrudability describes the ability of food materials to flow through a nozzle, whereas shape stability is the ability of food materials to form filaments, build the shape layer-by-layer, and maintain the shape after completion [12,13]. These characteristics are influenced by the rheological and mechanical properties of food materials. Rheological properties refer to mechanical properties that result in the deformation and flow of material in the presence of stress [14]. Liu et al. [13] studied the relationship between the rheology and printability of a food system composed of carrageenan, xanthan, and starch during a syringe-based 3DFP process that was divided into three stages, and the corresponding rheological properties of the food systems at the extrusion, recovery, and self-supporting stages were determined. Viscosity, yield stress, complex modulus, and shear-thinning behavior significantly affected the printability of food systems prepared at the extrusion and self-supporting stages. Umeda et al. [12] analyzed the printability of pumpkin paste from the viewpoint of fluid and structural mechanics using a screw-based 3D food printer and reported that extrudability is mainly affected by the loss tangent (tan δ) of the paste and balance between apparent viscosity and inner nozzle pressure, whereas the main factors for shape stability immediately after printing are Young’s modulus and the balance between stress applied to the printed food owing to its weight and yield stress of the paste.

In contrast, the mechanical properties are defined by [15] as the physical characteristics that arise from structural elements of foods, are sensed primarily by touch, are related to deformation, disintegration, and flow of the food under a force, and are measured objectively as functions of mass, time, and distance. Some mechanical properties of food include hardness, cohesiveness, adhesiveness, springiness, fracturability, chewiness, and gumminess. Tokuyasu et al. [16] found that the addition of Nata puree, prepared by the disintegration of Nata de coco (bacterial cellulose gel) with a water-soluble polysaccharide using a household blender, to potato paste increased its hardness and improved its shape retention during screw-based 3DFP.

The number of studies focusing on the printability of food materials has significantly increased. Examples include chocolate [17]; baking dough composed of water, sucrose, butter, wheat flour, and eggs [18]; mashed potatoes [19]; a mixture of hydrocolloids and broccoli, spinach leaves, and carrot powders [20]; egg yolk; and egg white with blends of rice flour [21]. However, to the best of our knowledge, the printability of nixtamalized corn flour (NCF) using screw-based 3DFP has not yet been investigated. NCF is highly important in Mexico and some other Latin American countries. The industrial production of NCF is based on the traditional method of nixtamalization, where maize kernels are cooked in a solution of Ca(OH)_2_ for 1 h and steeped in the cooking water with subsequent washing and grinding to produce a soft dough called masa that is referred to as nixtamalized corn dough (NCD) [22,23]. Nowadays, this process has been systemized for large-scale production, involving steps like cooking the maize kernels with lye and grinding the cooked grain to produce a dough that is dehydrated, sifted, classified, and packaged, obtaining NCF [24,25]. Afterwards, NCF is re-hydrated to obtain NCD. NCD is a soft dough that owes its color to the type of corn from which it is made (yellow, white, blue, red, etc.). Furthermore, due to its versatility, it is used as a raw material to produce tortillas and other corn-based products, such as soups, tamales, and drinks. Since NCD needs to be cooked to become edible, NCD products are submitted to different cooking processes, such as griddling, steaming, frying, or baking. The per capita consumption of corn tortillas in Mexico is 75 kg/year, demonstrating its economic, social, and cultural value to the Mexican population [26].

Depending on these data, this study aims to investigate the printability of NCF by correlating the rheological and mechanical properties of NCD with printing behavior at different phases of the screw-based 3DFP process. Because screw-based 3DFP represents a more complex mechanism than other extrusion methods, this study aims to understand the rheological and mechanical properties governing printability during this process. Furthermore, the use of NCF as a food ingredient for the 3DFP process might represent a convenient innovation because, until now, the only studied corn-based food is corn starch [27,28].

## 2. Materials and Methods

### 2.1. NCF

Blue corn-based NCF containing 8.0 g/100 g protein, 4.4 g/100 g lipid, and 63.0 g/100 g carbohydrate was purchased from Sodif S.A. de C.V. (Querétaro, Mexico).

### 2.2. Preparation of NCD Samples

NCD was prepared by mixing NCF and filtered tap water. First, NCF was weighed in a stainless-steel bowl, and filtered tap water at 25 °C was added. Subsequently, they were thoroughly mixed with a cooking spatula until a homogenized mixture was obtained. NCD was kept in a resealable plastic bag within an incubator at 25 °C until its utilization in the screw-based 3DFP process or its rheological and mechanical characterization on the same day. Seven NCDs were prepared using different weight ratios of NCF and water (Table 1) ranging from 25 to 40 wt% of NCF.

### 2.3. Screw-Based 3DFP

**Figure 1:**
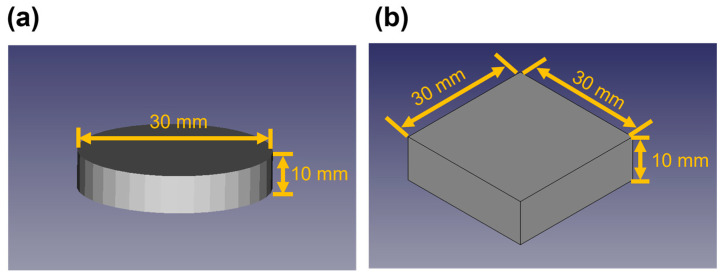
The designed three-dimensional (3D) models: (**a**) cylinder; (**b**) square pillar.

First, two different 3D models (Figure 1) were generated using FreeCAD v.0.19. The models consisted of a cylinder with a diameter of 30 mm and a height of 10 mm (Figure 1a) and a square pillar with a length of 30 mm and a height of 10 mm (Figure 1b). The .stl files of each model were exported and transferred to the Slicer software Slic3r v.1.2.9 to select printing parameters and generate their G-codes. The printing parameters are listed in Table 2. The inner diameter of the nozzle was 2.0 mm. The initial nozzle height was also set to 2.0 mm to equalize it with the inner nozzle diameter. The printing speed, which corresponds to the speed at which the moving stage shifts, was set to 20 mm/s. The extrusion multiplier specifies the rate at which the 3D food printer extrudes food materials by increasing or decreasing its screw rotation. This parameter was set to 2.0 as a result of previous trials aimed at matching, to some extent, the extrusion rate and printing speed. This corresponded to an actual screw rotation speed of approximately 2.6 rad/s [12]. The infill pattern was set as Concentric, and the number of outer shells was set to two. The infill percentage was set to 100%.

**Figure 2:**
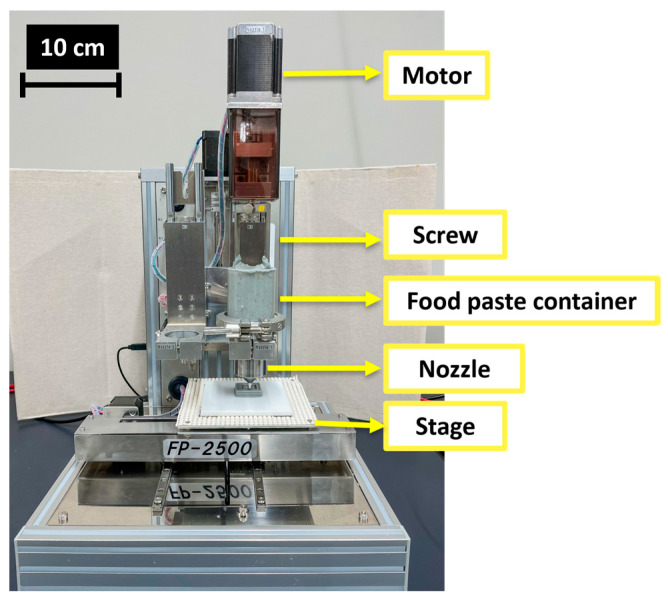
Photograph of the 3D food printer used in this study.

Subsequently, the generated G-codes were opened using the Pronterface v.1.6.0 software, which is used as a host of graphical user interfaces for 3D printing [29]. Finally, from the Pronterface v.1.6.0 software, 3D models were printed using a screw-based 3D food printer (FP-2500, Seiki Co., Ltd., Yamagata, Japan) equipped with an extruder (nozzle) moved by a motor (Figure 2). Each food paste container was filled to the top with the NCDs listed in Table 1. Each 3D model was printed in triplicate, following the parameters listed in Table 2. A room temperature of 25 °C was maintained during the whole screw-based 3DFP process, and no heating was applied in the 3D food printer.

### 2.4. Weight and Dimensional Analysis

**Figure 3:**
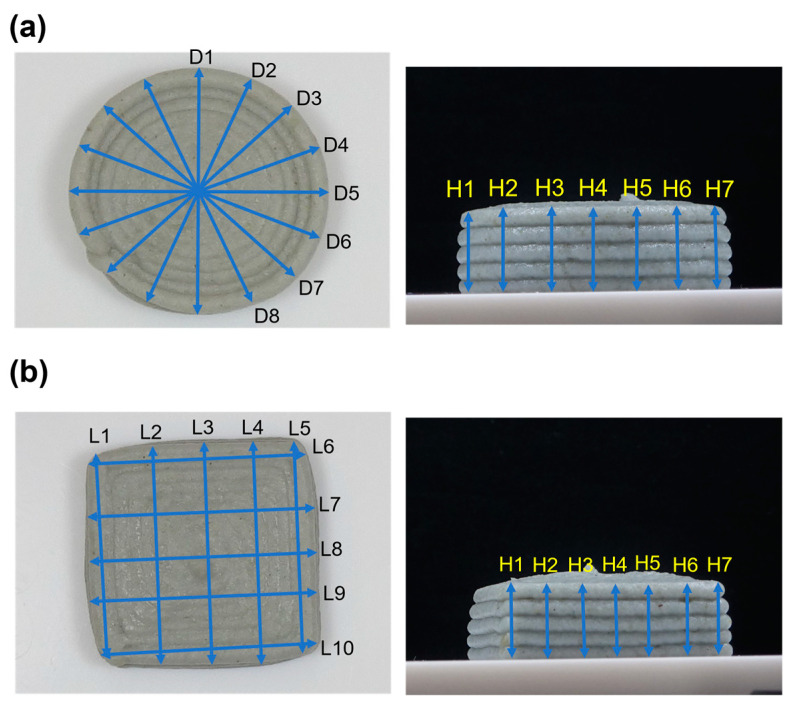
Points of size measurements of the 3D-printed foods: (**a**) cylinder, diameter, and height; (**b**) square pillar, length, and height.

To assess the print quality of the 3D-printed foods, they were weighed and photographed within approximately 5 min after printing [12]. The weight of each 3D-printed food item was measured using a weighing balance, and the average and standard deviation were calculated using data from the three replicas for each 3D model. To analyze the accuracy of the 3D-printed foods, i.e., to assess the similarity between the sizes of printed samples and designed models, ImageJ v.1.53t (National Institutes of Health, Bethesda, MD, USA) was used to obtain the size measurements of each 3D-printed food item. The diameters of the cylinders (Figure 3a) were measured at eight different points in the top view, and the average was reported as the diameter of the printed cylinder. Furthermore, their heights were measured at seven different points in the side view, and the average was reported as the height of the printed cylinder. The lengths of square pillars (Figure 3b) were measured at ten different points in the top view, and the average was reported as the length of the printed square pillar. In addition, their heights were measured at seven different points in the side view, and the average was reported as the height of a printed square pillar. The acceptable ranges for the measured height and diameter were defined as ±10% of the designed size.

### 2.5. Rheological Analysis

Rheological characterization of the NCDs was performed using a dynamic rheometer (HAAKE MARS iQ Air; Thermo Fisher Scientific Inc., Waltham, MA, USA) equipped with a plate–plate geometry (35 mm diameter; 1 mm gap) at 25 °C. Four different experiments were performed: (1) rotational ramp, (2) oscillation strain sweep, (3) oscillation stress sweep, and (4) oscillation frequency sweep. Each experiment was performed in triplicate, and the average data were used to plot the graphs. Please see Supplementary Materials for more information on oscillation frequency sweep.

#### 2.5.1. Rotational Ramp

A rotational ramp experiment was conducted in flow–curve mode to study the flow behavior of the prepared NCDs. The apparent viscosity and shear stress were obtained at a shear rate increasing from 0.01 to 100 s^−1^ logarithmically in 90 s. To determine the apparent viscosity, the shear stress was divided by the shear rate in each measurement according to the following equation:

(1) $$\eta =\frac{\tau }{\dot{y}}$$

where τ is the shear stress, ẏ is the shear rate, and η is the apparent viscosity.

#### 2.5.2. Oscillation Strain Sweep

An oscillation strain sweep was used to study the responses of the elastic modulus G′ and viscous modulus G″ to strain. This test was performed at a strain range of 0.0003–100 and a constant frequency of 1 Hz. The linear viscoelastic region (LVR) of the NCDs was determined, and the critical strain at which the LVR was finished corresponded to a 10% drop in the plateau of G′. Additionally, the loss tangent at nozzle extrusion strain (tan δ_yE_) was obtained using the following equation:

(2) $${\tan\delta }_{\mathrm{yE}}=\frac{{G^{\prime \prime }}_{\mathrm{yE}}}{{G^{\prime }}_{\mathrm{yE}}}$$

where G″_yE_ and G′_yE_ are the viscous and elastic moduli at nozzle extrusion strain, respectively. The nozzle extrusion strain was considered to be 14, according to Umeda et al. [12]. Equation (2) was used to obtain the loss tangent at minimum strain (tan δ_min_). However, the values of G″_yE_ and G′_yE_ were substituted by G″_min_ and G′_min_, which corresponded to the viscous and elastic moduli at a minimum strain, respectively. The minimum strain was considered to be 0.0003, which was the first strain value measured using a rheometer.

#### 2.5.3. Oscillation Stress Sweep

The responses of G′ and G″ to stress were measured using an oscillation stress sweep test. A stress range of 1–1000 Pa and a constant frequency of 1 Hz were used. The yield stress of each NCD was determined as the shear stress at the crossover point where G′ is equal to G″ [30].

### 2.6. Analysis of Texture Profiles

Texture profile analysis (TPA) was performed using a Texture Profile Unit (TPU-2D; Yamaden Co., Ltd., Tokyo, Japan). First, 28 g of each prepared NCD was placed in a container with an inner diameter of 48 mm and a height of 15 mm. Subsequently, the filled container was placed in the Texture Profile Unit to perform TPA under the following conditions: a 16 mm diameter plunger was used to compress the sample at a compression speed of 10 mm/s, clearance of 5 mm, and two measurement cycles using the constant-speed compression method. The temperature of the samples was 25 °C. Ten measurements were performed for each NCD, and averages and standard deviations were reported for the hardness, adhesiveness, and cohesiveness of the samples.

## 3. Results

### 3.1. Screw-Based 3DFP

**Figure 4:**
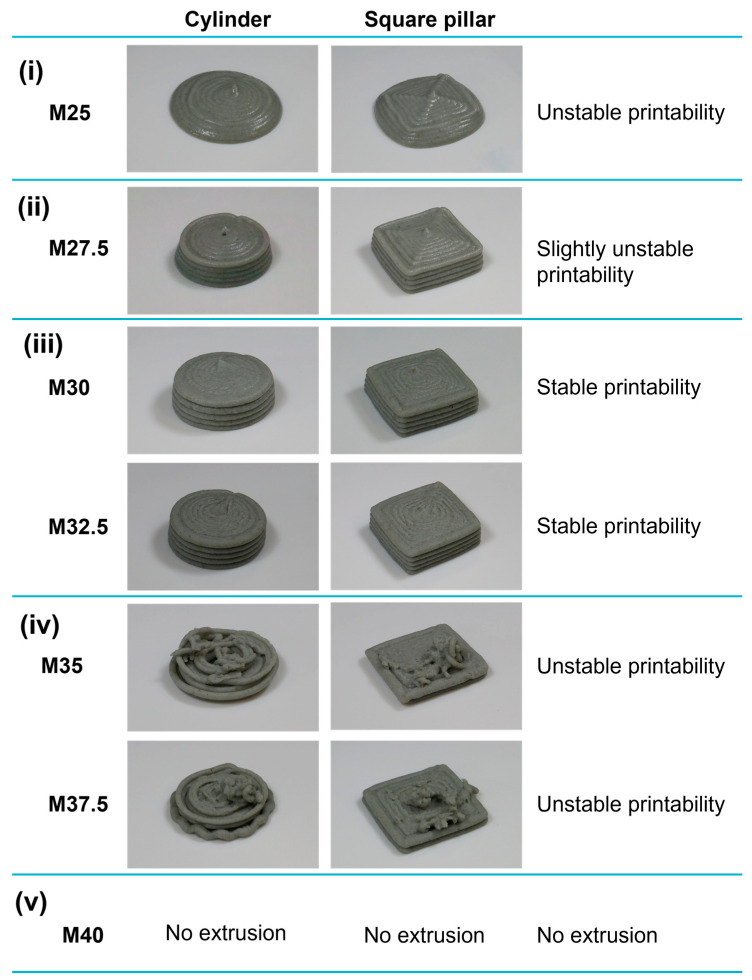
Photographs of the 3D-printed foods: (**i**) unstable printability at 25 wt% of nixtamalized corn flour (NCF); (**ii**) slightly unstable printability at 27.5 wt% of NCF; (**iii**) stable printability between 30.0 and 32.5 wt% of NCF; (**iv**) unstable printability between 35.0 and 37.5 wt% of NCF; and (**v**) no extrusion at 40.0 wt% of NCF.

**Figure 5:**
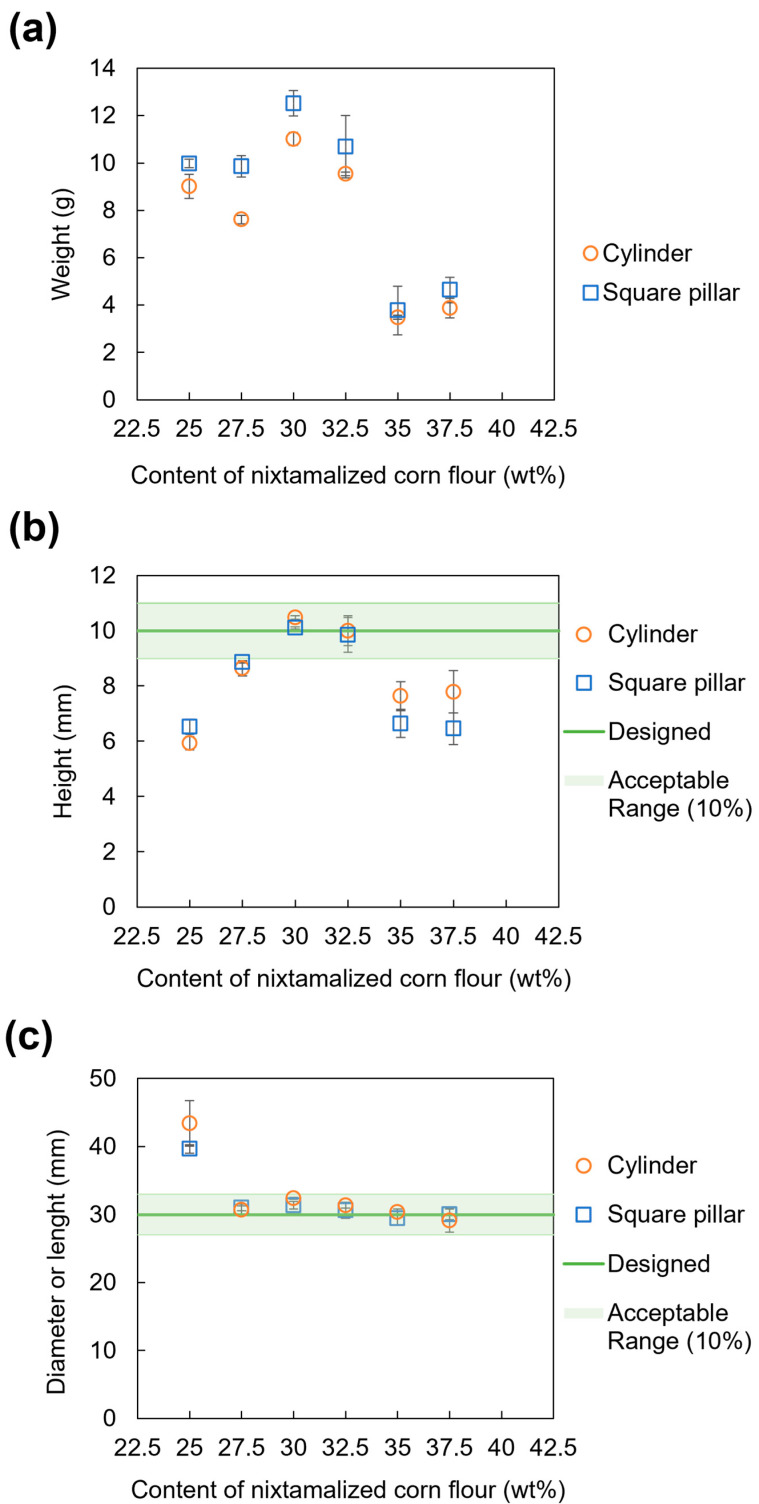
Weight and dimensional analysis of the 3D-printed foods: (**a**) weight; (**b**) height; and (**c**) diameter of cylinder or length of square pillar.

The results of screw-based 3DFP experiments are shown in Figure 4. Additionally, Figure 5 shows the weight, height, and diameter or length of 3D-printed foods for each NCF level. In general, Figure 4 can be divided into five regions (i–v): The first region has an NCF content below 25.0 wt% (Figure 4i). In this region, extrusion of the NCD was possible. However, unstable shape retention and high deformation of 3D-printed foods were observed. The second region has an NCF content of 27.5 wt% (Figure 4ii). Extrusion of the NCD was continuous. However, shape formation and retention were slightly unstable. The third region has an NCF content of 30.0 to 32.5 wt% (Figure 4iii). In this region, extrusion of the NCD was continuous, and stable shape formation and retention were observed. The fourth region has an NCF content > 32.5 wt%. In this region, noncontinuous extrusion of the NCD was observed at 35 and 37.5 wt% of NCF (Figure 4iv). Therefore, they were not capable of forming 3D models and exhibited unstable shape formation. In the fifth region, extrusion of the NCD was not possible when an NCF content of 40 wt% was used (Figure 4v).

The weights of 3D-printed foods (Figure 5a) in the first and second regions were similar, ranging between approximately 8 and 10 g. Subsequently, the weight of 3D-printed foods in the third region increased to approximately 9–13 g. In contrast, the weight of 3D-printed foods in the fourth region drastically decreased because of unstable extrusion of the NCD, where the 3D models were incompletely formed.

Compared to the size of designed 3D models, the low height and large diameter of 3D-printed foods in the first region (Figure 5b,c) confirmed unstable shape retention and high deformation. However, although the length of 3D-printed foods in the second region (Figure 5c) was within the acceptable range (±10% of the designed size), their height (Figure 5b), which was slightly below the acceptable range, confirmed that the NCD with 27.5 wt% NCF was not capable of maintaining the shape after printing. In contrast, the height and length of 3D-printed foods in the third region (Figure 5b,c) validated continuous extrusion, stable shape formation, and retention since these values were very close to those of the designed 3D models, within the acceptable range of ±10% of the designed dimensions. The height of 3D-printed foods (Figure 5b) in the fourth region drastically decreased because of the lack of NCD in the top layers. This behavior confirmed the noncontinuous extrusion of the NCD within the fourth region.

In summary, stable extrudability and shape retention can be achieved using NCD with NCF content ranging from 30 to 32.5 wt%.

### 3.2. Rheological Properties

#### 3.2.1. Flow Behavior and Apparent Viscosity

**Figure 6:**
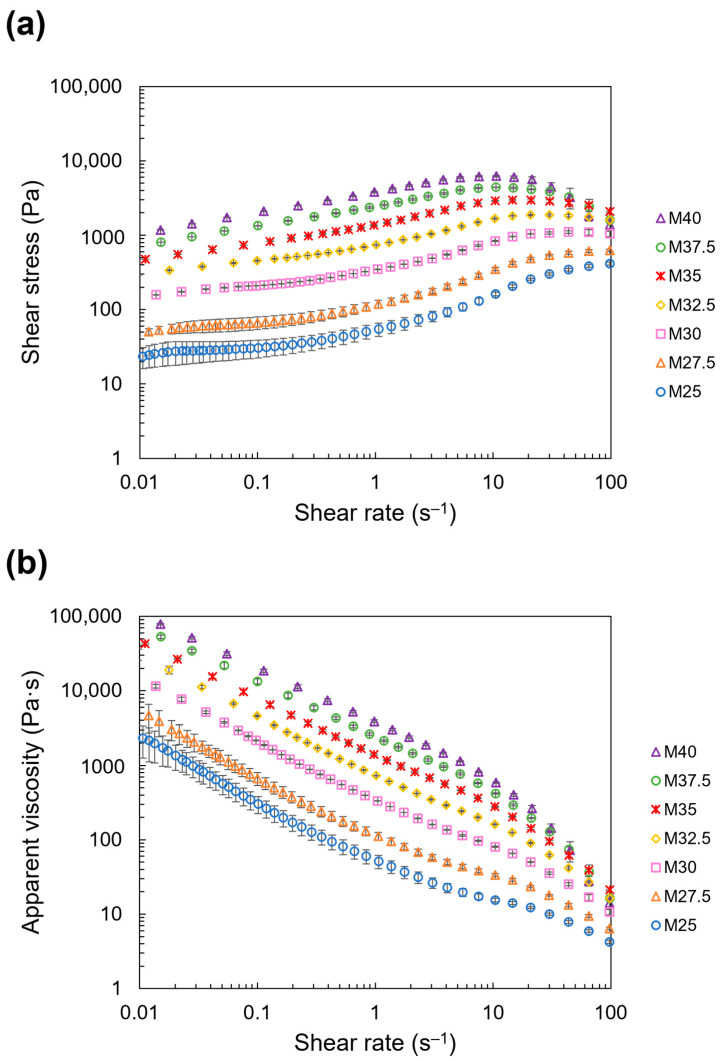
Dependence of shear stress and apparent viscosity of the NCD on applied shear rate: (**a**) shear rate; (**b**) apparent viscosity (plots and error bars show the mean and standard deviation of three repeated measurements, respectively).

Flow curves demonstrating the dependence of shear stress and apparent viscosity on the applied shear rate during the rotational ramp test are shown in Figure 6a,b. An increase in the shear rate resulted in an increase in the shear stress (Figure 6a) and a decrease in the apparent viscosity (Figure 6b). These profiles are characteristic of shear-thinning behavior, where viscosity decreases with an increase in the shear rate [31]. In contrast, the shear stress and apparent viscosity (Figure 6a,b) increased with the addition of NCF at a specific shear rate.

#### 3.2.2. Elastic and Viscous Moduli

**Figure 7:**
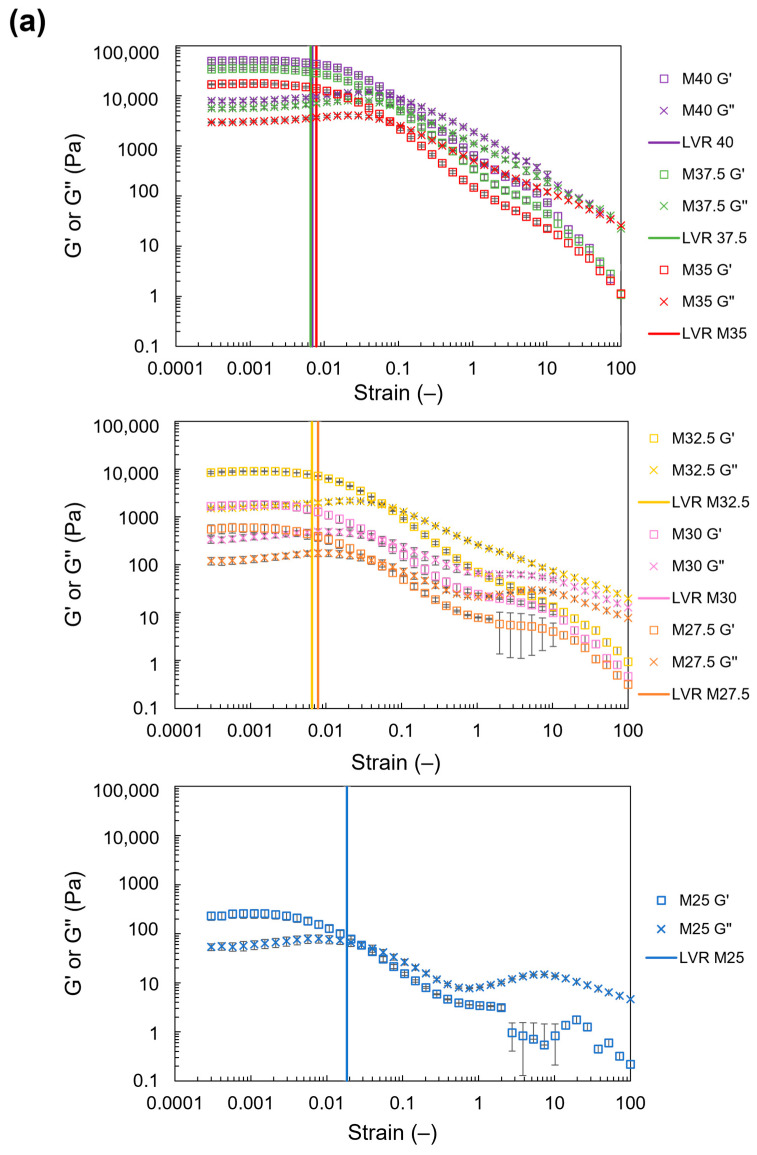

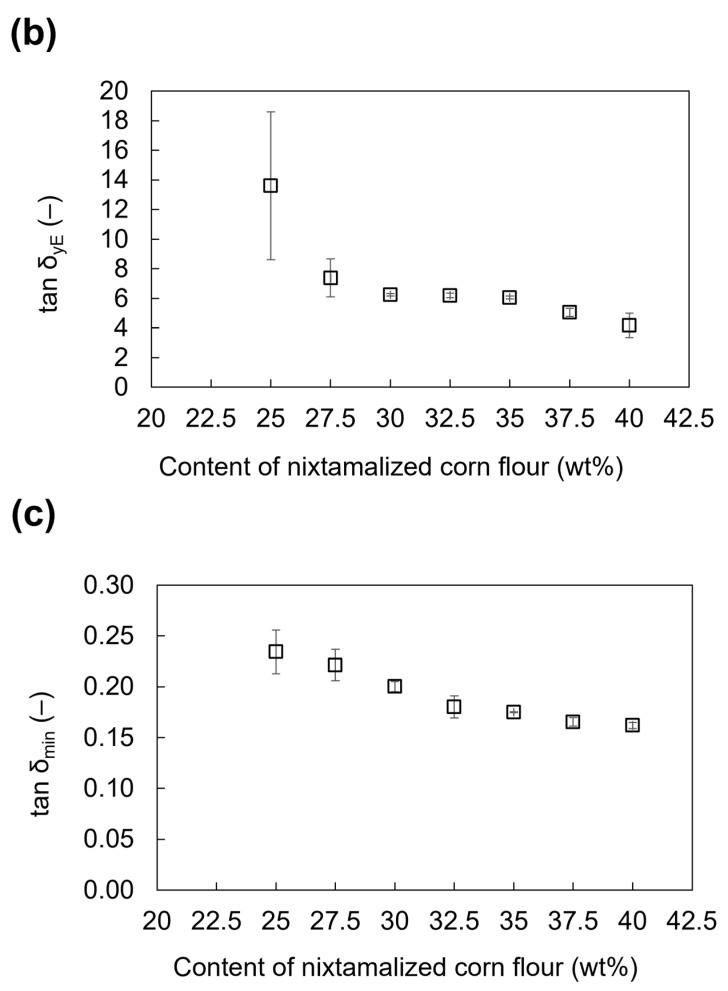
(**a**) The effect of strain on G′ and G″ of the NCD in its linear viscoelastic region (LVR); (**b**) the effect of NCF content on the loss tangent at nozzle extrusion strain; and (**c**) the effect of NCF content on the loss tangent at minimum strain (plots and error bars show the mean and standard deviation of three repeated measurements, respectively).

**Figure 8:**
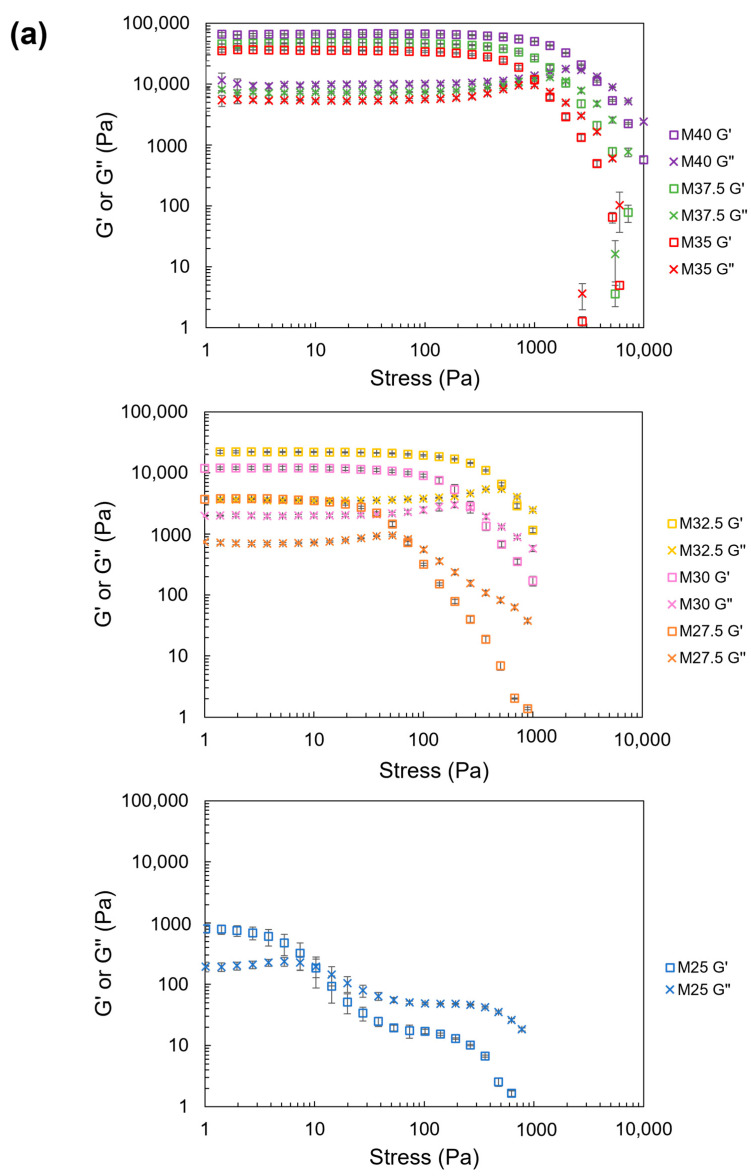

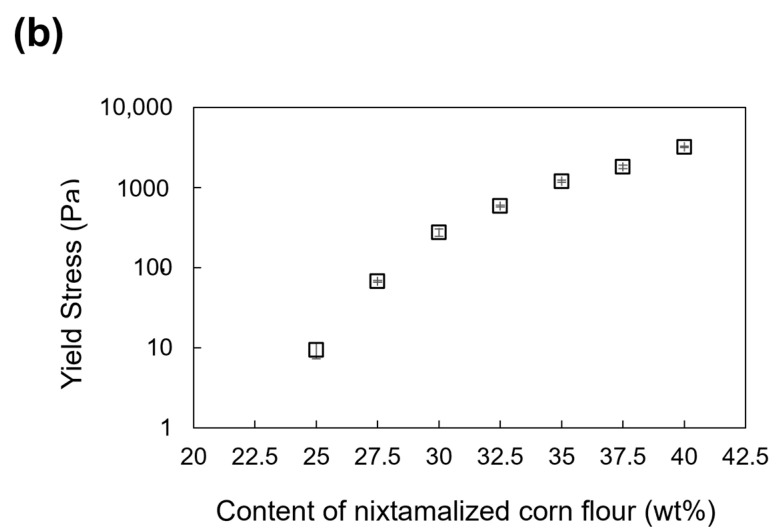
(**a**) The effect of shear stress on G′ and G″ of the NCD; (**b**) the effect of NCF content on the yield stress (plots and error bars show the mean and standard deviation of three repeated measurements, respectively).

The results of the oscillation strain sweep (Figure 7a) and oscillation stress sweep (Figure 8a) showed that the values of G′ and G″ increased at high NCF contents. This indicates that a high mechanical strength was observed in samples with a high NCF content. Additionally, for all NCDs, G′ was >G″. However, with an increase in stress or strain, G′ decreased until reaching a cross-point between G′ and G″ for all NCDs. Because G′ represents the solid-like behavior, and G″ represents the fluid-like behavior of materials, a cross-point between these two values indicates that the fluid-like behavior becomes dominant. The cross-point between G′ and G″ in the oscillation stress sweep represents the yield stress of NCDs [30]. The results showed that the yield stress increased with a high content of NCF (Figure 8b) from 9.4 Pa in M25 (25 wt% of NCF) to 3220.0 Pa in M40 (40 wt% of NCF). An analysis of the time dependence of G′ and G″ is presented in the Supplementary Materials.

#### 3.2.3. Loss Tangent

The values of tan δ_min_ and tan δ_yE_ were estimated from the results in Figure 7a. Both tan δ_yE_ and tan δ_min_ decreased at high contents of NCF (Figure 7b,c, respectively). The values of tan δ_yE_ decreased from 13.6 in M25 to 4.1 in M40, while the values of tan δ_min_ decreased from 0.23 in M25 to 0.16 in M40. A detailed analysis of the LVR of the NCDs and their critical strains is presented in the Supplementary Materials.

### 3.3. Texture Properties

**Figure 9:**
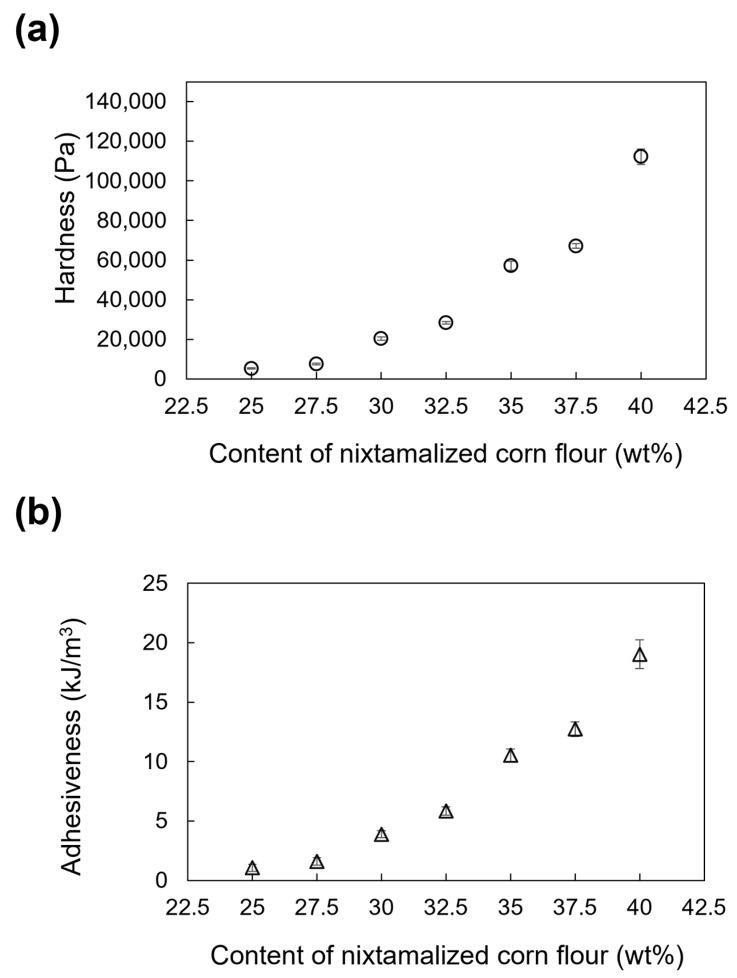

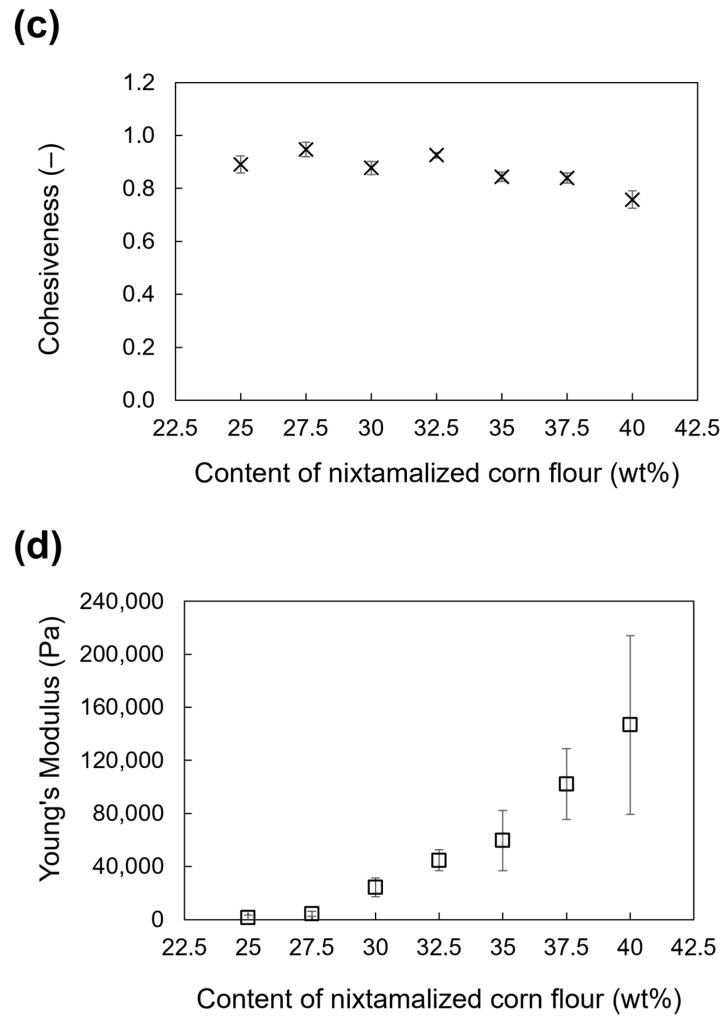
Effect of NCF content on (**a**) hardness; (**b**) adhesiveness; (**c**) cohesiveness; and (**d**) Young’s modulus of the nixtamalized corn dough (plots and error bars show the mean and standard deviation of 10 repeated measurements, respectively).

Figure 9 shows the hardness, adhesiveness, cohesiveness, and Young’s modulus of the NCDs. The hardness, adhesiveness, and Young’s modulus increased at high contents of NCF. The hardness increased from 5259.71 Pa in M25 to 112,229.61 Pa in M40; adhesiveness increased from 1.06 kJ/m^3^ to 19.03 kJ/m^3^ in M25 and M40, respectively; and Young’s modulus increased from 1538 Pa in M25 to 146,729 Pa in M40. However, a major change in the cohesiveness of NCDs was not evident because it fluctuated with the NCF content, with a minimum value of 0.76 in M40 and a maximum value of 0.95 in M27.5 (27.5 wt% of NCF). Cohesiveness may be an independent variable of NCF content. Similar results, except for cohesiveness, have been obtained by Umeda et al. [12], who observed that pumpkin paste showed a decrease in cohesiveness with a decrease in pumpkin flake content.

## 4. Discussion

**Figure 10:**
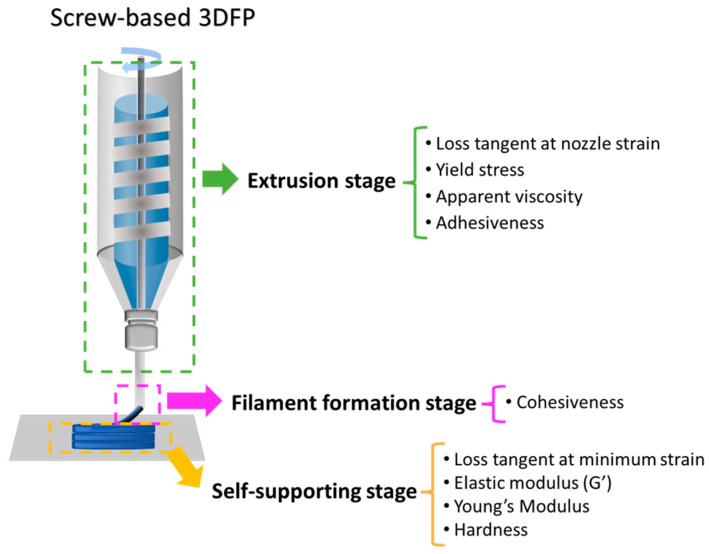
Relationship between printability of the nixtamalized corn dough during screw-type 3D food printing (3DFP) and its rheological and mechanical properties.

To correlate the rheological and mechanical properties of NCDs with their printability, the screw-based 3DFP process was divided into three stages: extrusion, filament formation, and self-support (Figure 10). Additionally, the corresponding rheological and mechanical properties of NCDs at each stage were discussed. The diagram in Figure 10 is based on the relationship between printability and rheological properties of food materials during syringe- and screw-based 3DFP established by Liu et al. [13] and Umeda et al. [12], respectively.

### 4.1. Extrusion Stage

Because the loss tangent (tan δ) is the ratio of G″ and G′, the analysis of dynamic behaviors of G′ and G″ at different strain values becomes easier. In principle, if tan δ > 1, G″ is dominant, and the material exhibits mainly viscous behavior; in contrast, if tan δ < 1, the material exhibits mainly elastic behavior [29]. In this study, tan δ_yE_ decreased with an increase in the NCF content. This indicates that the viscous behavior at nozzle extrusion strain was high in M25 and decreased with the addition of NCF. In any case, tan δ_yE_ was >1 in all NCDs, indicating that a viscous behavior was dominant at nozzle extrusion strain. Therefore, they had the potential to behave as fluids during extrusion.

The yield stress of samples is defined as the stress required for G′ to exceed G″ and is closely related to the minimum pressure required to initiate the flow of food materials [13,32]. During extrusion-based 3DFP, extrusion of the food material is not continuous and frequently starts and stops according to the movements of the 3D food printer and the formation of the layers. Therefore, food materials should have an appropriate yield stress that allows continuous extrusion. In this study, a high-yield stress was obtained at high NCF contents. Therefore, M40 required more pressure for extrusion than M30 (30 wt% of NCF) and M25. This was confirmed by the results obtained in the screw-based 3DFP experiment, where noncontinuous extrusion was observed in the 3D-printed foods using M35 (35 wt% of NCF) and M37.5 (37.5 wt% of NCF) doughs. Moreover, M40 could not be extruded through the nozzle because it was above the threshold of extrudable yield stress. In contrast, continuous extrusion was observed in samples M25, M27.5, M30, and M32.5 (32.5 wt% of NCF), which exhibited low yield stress.

Once the yield stress is exceeded and food materials begin to flow, maintaining a continuous flow of food materials is necessary. The pressure required to maintain a constant flow depends on the viscosity and adhesiveness of samples [13]. Viscosity is the frictional force inside the dough, and adhesiveness is the frictional force between the dough and the nozzle wall. Umeda et al. [12] have demonstrated that the pressure in the nozzle of a screw-based 3D food printer increases at high apparent viscosity. In contrast, adhesiveness is defined in the TPA as the necessary work to pull the compressing plunger away from the sample and reflect the bonding power to a surface. A strong relationship exists between adhesion and friction. Solids with high friction coefficients typically exhibit strong adhesion properties. The pressure required to extrude food materials increases with the force necessary to overcome friction between the food materials and the walls of the food paste container. This means that high pressure is required for extrusion in case of high adhesiveness [33,34]. The apparent viscosity and adhesiveness of the NCDs increased with the addition of NCF. Therefore, relatively low pressure was required to extrude M25, M27.5, M30, and M32.5. In contrast, relatively high pressure was required to extrude M35, M37.5, and M40. This was confirmed through the screw-based 3DFP process, where continuous extrusion was observed in M25, M27.5, M30, and M32.5, while samples with very high apparent viscosity and adhesiveness, such as M35 and M37.5, exhibited noncontinuous extrusion. This indicates that high apparent viscosity and adhesiveness make extrusion challenging.

### 4.2. Filament Formation Stage

Cohesion is the inner strength of a material that must withstand external forces without breaking [35]. It is determined by the intermolecular attraction by which the elements of a body or mass material are held together. This study hypothesized that cohesiveness might affect filament formation during the extrusion-based 3DFP process because high cohesiveness could result in a continuous filament, whereas the filaments would break at low cohesiveness. However, the results did not show a major change in the cohesiveness of NCDs, suggesting that cohesiveness might be independent of the NCF content. Further analysis of the tensile strength or surface tension, together with the pressure inside the nozzle and velocity of extrusion, might help better explain the filament-formation stage. Umeda et al. [12] have reported that the cohesiveness of pumpkin paste reduces to 0.6–0.7 at low pumpkin flake content; however, the 3D-printed foods generated using low-cohesiveness pumpkin paste present high weight. Therefore, thick filaments may be formed at low cohesiveness, causing an increase in weight. In contrast, in this study, the cohesiveness of NCDs was almost the same in all NCF contents. Furthermore, no increase in the weight of 3D-printed foods at low NCF contents (M25 and M27.5) was observed. Therefore, extruded filaments might be sufficiently cohesive to maintain filament diameters, even at low NCF contents. This trend may be unique to NCD.

### 4.3. Self-Supporting Stage

Because the values of tan δ_min_ were <1, the solid-like behavior (G′) of all the NCDs was expressed more than the fluid-like behavior (G″) in the static state. Additionally, tan δ_min_ decreased at high NCF contents, indicating that the solid-like behavior was more dominant at NCF contents > 30 wt%.

G′ represents the dynamic modulus of elasticity, while Young’s modulus represents the static elastic modulus of the NCDs. These parameters can reflect the solid-like behavior and mechanical strength of NCDs, thereby influencing the capability of food materials to maintain the printed shape after extrusion. Additionally, hardness is the ability of a material to resist deformation, and it increases in correlation with Young’s modulus [12,36,37]. The values of G′, Young’s modulus, and hardness increased at high NCF contents. For this reason, the figures printed with M25 and M27.5 could not retain the shape, whereas those with M30 and M32.5 exhibited stable shape retention. However, although M35 and M37.5 seemed to retain the shape after formation owing to high G′, Young’s modulus, and hardness, the previous extrusion process was not continuous. Consequently, shape formation was incomplete.

In addition to the abovementioned mechanical properties, the yield stress may also have an effect. The yield stresses of M25 and M27.5 were very low. Furthermore, these NCDs exhibited unstable shape retention. This suggests that the stress applied to 3D-printed food owing to its weight exceeded the yield stress, resulting in its fluidization and flattening [12].

### 4.4. General Discussion and Future Prospects

Stable printability was obtained at a specific range of NCF content in the NCD. Similar results have been observed for other food pastes, such as pumpkin paste, where the yield stress, apparent viscosity, and other rheological and mechanical properties were affected by the flake content of pumpkin paste [12]. However, one main difference between the results reported by Umeda et al. [12] and the results obtained in this study is that in the former, the cohesiveness was affected by the flake content in the pumpkin paste, which affected the weight of the 3D-printed foods. In contrast, in this study, the cohesiveness of the NCD was not affected by the NCF content, implying that the weight difference observed in the 3D-printed foods is caused by the changes in the properties involved in the extrusion stage (tan δ_yE_, yield stress, apparent viscosity, and adhesiveness), not by the properties of the filament formation stage (cohesiveness). This may be a unique trend for NCD. Furthermore, stable printability of NCD was achieved by mixing water and NCF without adding any other ingredient or additive, suggesting that NCD has a noncomplex composition that can be used in extrusion-based 3DFP.

A more detailed analysis of the extrusion stages, considering the pressure inside the nozzle and the velocity of the extrusion, may help better understand the behavior of NCD inside the nozzle. Additionally, during filament formation, the cohesiveness was not affected by NCF content in the NCDs, suggesting that the mechanism of filament formation should be analyzed in more detail. This analysis can include other characteristics such as tensile strength, surface tension, shear recovery, and the previously mentioned analysis of the pressure and extrusion velocity inside the nozzle. Regarding the self-supporting stage, a dimensional analysis of the 3D-printed foods was performed using 2D cross-sections In addition, a more accurate analysis of the 3D shape can help better evaluate shape formation and stability.

This study is the first step in using NCD to produce 3D-printed foods. Further research on the development of 3D-printed foods made of NCD, such as the effect of post-printing methods, like cooking processes, on the final characteristics of the 3D-printed foods, is needed.

## 5. Conclusions

In this study, the printability of NCD was investigated by correlating its rheological and mechanical properties with its printing behavior during screw-based 3DFP. Stable printability was obtained at a specific range of NCF content in the NCD. Below this NCF content, the 3D-printed foods flattened, while above this NCF content, the extrudability of the paste was affected. It was clarified that the printability of NCD is influenced by different rheological and mechanical properties, depending on the stages of the screw-type 3DFP process. During the extrusion stage, tan δ_yE_, yield stress, apparent viscosity, and adhesiveness mainly affected the extrudability of the NCD. In contrast, tan δ_min_, G′, Young’s Modulus, and hardness were found to influence the self-supporting stage. Therefore, it is important to find a balance between all these properties, where stable extrudability and self-supporting of the 3D structure are achieved. This study can provide guidance for evaluating the printability of other food materials intended for screw-based 3DFP. Furthermore, this study represents a first step in producing 3D-printed foods using NCD.

## Figures and Tables

**Table 1:** Composition of the nixtamalized corn dough (NCD).

Symbol	Nixtamalized Corn Flour Content (wt%)	Purified Water Content (wt%)
M25	25.0	75.0
M27.5	27.5	72.5
M30	30.0	70.0
M32.5	32.5	67.5
M35	35.0	65.0
M37.5	37.5	62.5
M40	40.0	60.0

**Table 2:** Printing parameters.

Parameter	Condition
Print speed (stage movement speed) (mm/s)	20
Nozzle inner diameter (mm)	2.0
Initial nozzle height (mm)	2.0
Extrusion multiplier (-)	2.0
Infill pattern (-)	Concentric
Infill percent (%)	100
Number of outer shells (-)	2
Temperature (°C)	25

## Data Availability

The data presented in this study are available in the Supplementary material section. Data will be made available on request from the corresponding author.

## Supplementary Materials

The following supporting information can be downloaded at https://www.mdpi.com/article/10.3390/foods13020293/s1. Text 1: Linear viscoelastic region (LVR) [38,39]; Text 2: Frequency-dependence of G′ and G″.
