# Supplementary material for: Printability of Nixtamalized Corn Dough during Screw-Based Three-Dimensional Food Printing
Source: Foods. 2024 Jan 17;13(2):293. doi: 10.3390/foods13020293 (PMC10815360; doi:10.3390/foods13020293)
Supplement: Supplementary file 1 [file foods-13-00293-s001.zip › foods-2808729-supplementary.pdf]

foods

Supplementary materials

Text 1: Linear Viscoelastic Region (LVR)

Oscillation strain sweep was performed on the nixtamalized corn doughs (NCDs) to estimate their LVRs. LVR is the range of strain, where G′ and G′′ are constants. Within this region, a material can be deformed without breaking its internal structure, experiencing reversible deformation; however, when the material reaches a critical strain, structure of the material is irreversibly deformed [1]. No significant change in the critical strain of the NCDs was observed (Figure 7a), with values 0.007 in M27.5 and 0.006 in M40. These results indicate that the critical strain is independent of moisture content. Additionally, the strain–stress curves were obtained for each NCD (Figure s1). The stress required to reach the critical strain was obtained by measuring the stress at a given critical strain, as shown in Figure s1. The stress required to reach the critical strain increased at high NCF concentrations, as shown in Figure s2, from 1.8 Pa in M25 to 310 Pa in M40. This suggests that although the critical strain may be independent of the moisture content, the stress required to reach the specific critical strain depends on the moisture content.

Because G′ and G′′ are strain-independent within the LVR, and structural breakdown is not achievable, important microstructural properties, such as time dependency of G′ and G′′, can be measured [1, 2]. Therefore, the LVR was determined before the oscillation frequency sweep test. The experimental results (Figure 7a) showed that a strain of 0.1% (0.001 strain) was present inside the LVR of all NCDs. The strain was constant during the oscillation frequency test.

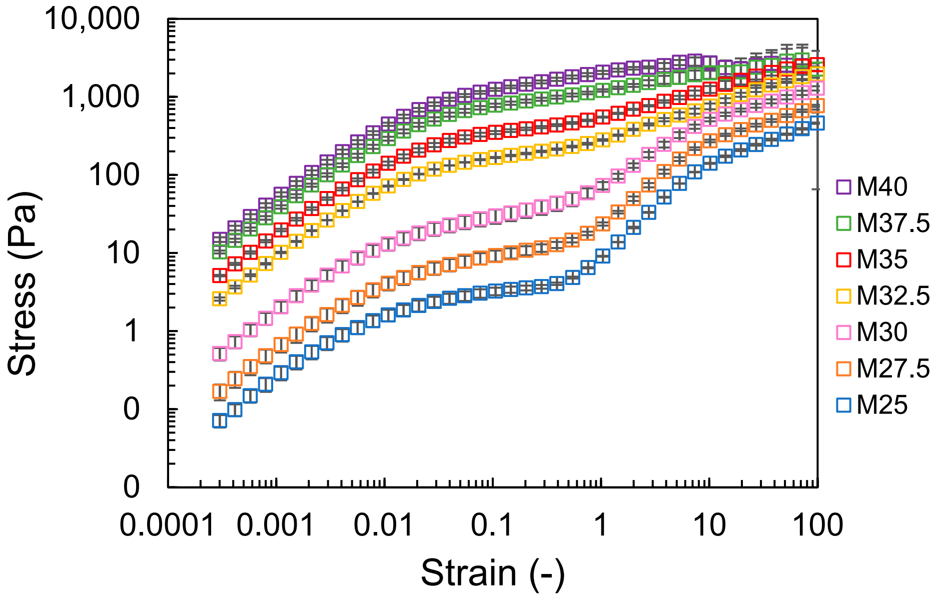

Figure S1. Strain-stress curves of the NCD (plots and error bars show the mean and standard deviation of three repeated measurements, respectively).

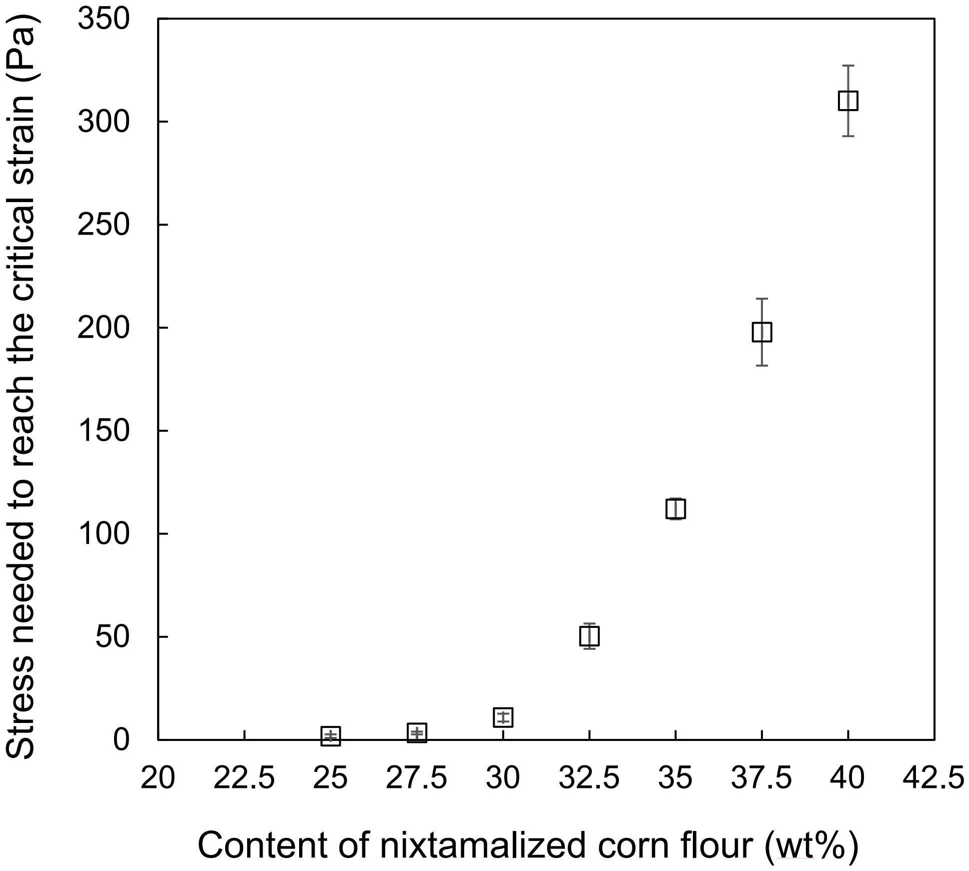

Figure S2. Stress needed to reach the critical strain at specific NCF concentrations (plots and error bars show the mean and standard deviation of three repeated measurements, respectively).

Text 2: Frequency-dependence of G′ and G′′

To determine the time dependency of G′ and G′′ of the NCDs, an oscillation frequency sweep analysis was performed at frequencies ranging between 0.1–10 Hz at a constant deformation of 0.1% strain (within the LVR previously determined through an oscillation strain sweep test). In this test, the oscillation frequency was ramped while the strain was held constant. The speed of deformation of the sample changed, while G′ and G′′ were monitored within the LVR. This test should be performed within the LVR of the samples to ensure that G′ and G′′ are independent of the strain and stress, and that their responses depend on the internal structure of the samples. This test method provides information about G′ and G′′. Because the inverse of frequency is time, frequency sweep tests were used to investigate time-dependent deformation. A high frequency corresponds to fast deformation, and a low frequency corresponds to slow deformation. Therefore, the frequency sweep data can be used to evaluate whether the response of a sample to a certain deformation speed is viscous or elastic. At a given frequency, if G′ is > G′′, the response of the sample to deformation is dominated by elasticity, and the sample behaves more elastically. If G′ is < G′′, the sample behaves more viscously [1].

The test results (Figure s3) showed that G′ was > G′′ at all frequencies studied for all NCDs. Therefore, the responses of the NCDs to deformation was dominated by elasticity, and the NCDs behaved more elastically. Furthermore, the time dependency of G′ and G′′ was minimal because their increments were small with an increase in the frequency.

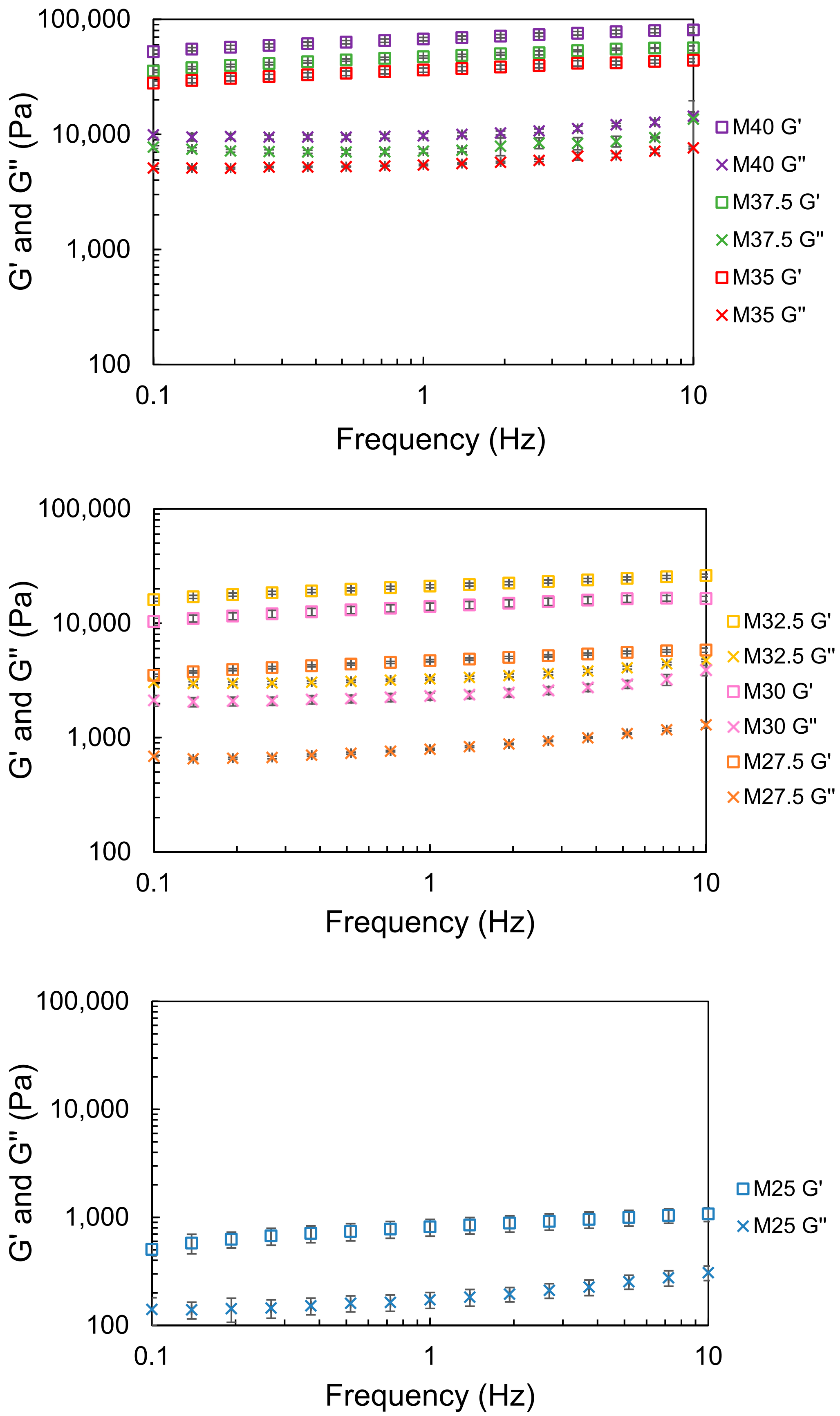

Figure S3. Effect of frequency on G′ and G′′ of the NCD (plots and error bars show the mean and standard deviation of three repeated measurements, respectively).

References

1. Bui, B.; Saasen, A.; Maxey, J.; Ozbayoglu, E.; Miska, S.; Yu, M. Viscoelastic properties of oil-based drilling fluids. *Annu. Trans. Nord. Rheol. Soc.* **2012**, *20*, 33–47.
2. TA Instruments. Available online: https://www.tainstruments.com/pdf/literature/RH107.pdf (accessed on 30 June 2023).
